# Geographic Availability of Low-Dose Computed Tomography for Lung Cancer Screening in the United States, 2017

**DOI:** 10.5888/pcd15.180241

**Published:** 2018-10-04

**Authors:** Jan M. Eberth, Parisa Bozorgi, Logan M. Lebrón, Sarah E. Bills, Linda J. Hazlett, Ruth C. Carlos, Jennifer C. King

**Affiliations:** 1 Department of Epidemiology and Biostatistics, Arnold School of Public Health, University of South Carolina, Columbia, South Carolina; 2 Cancer Prevention and Control Program, Arnold School of Public Health, University of South Carolina, Columbia, South Carolina; 3 South Carolina Rural Health Research Center, Arnold School of Public Health, University of South Carolina, Columbia, South Carolina; 4 Department of Environmental Health Sciences, Arnold School of Public Health, University of South Carolina, Columbia, South Carolina; 5 Department of Geography, University of South Carolina, Columbia, South Carolina; 6 Department of Psychology, University of South Carolina, Columbia, South Carolina; 7 Department of Radiology, University of Michigan, Ann Arbor, Michigan; 8 Lung Cancer Alliance, Washington, DC

**Figure:**
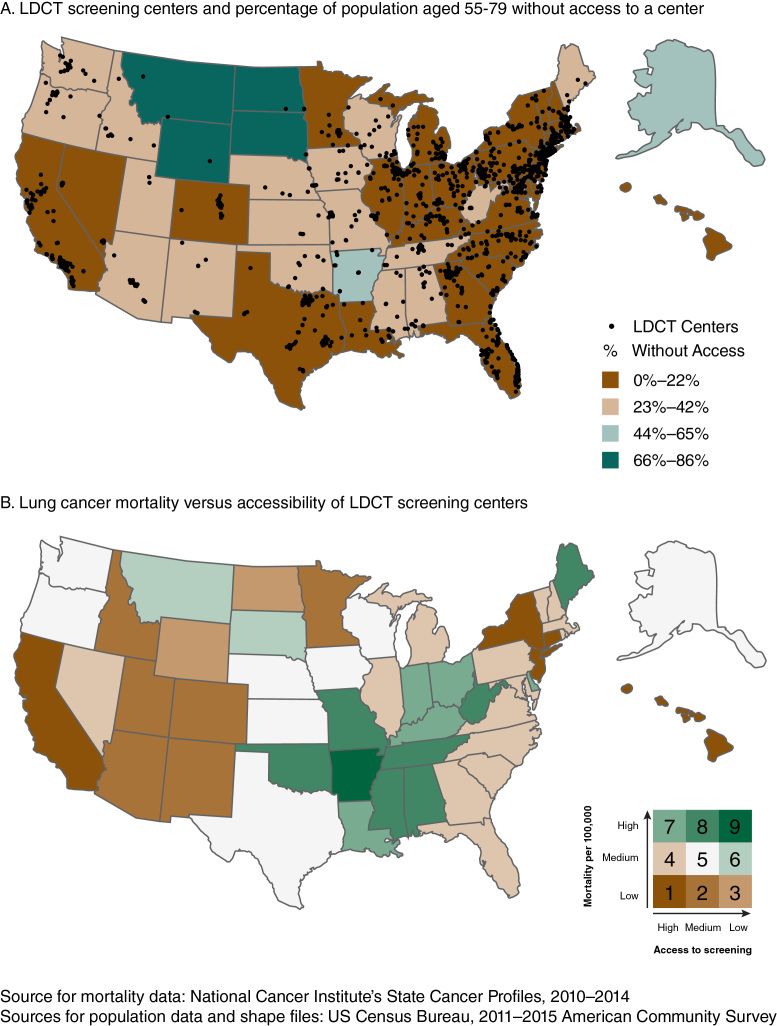
Panel A. Location of low-dose computed tomography (LDCT) screening centers in the United States and percentage of US population aged 55 to 79 who live without access to a screening center within 30 miles. Symbol indicates location of a LDCT screening center. Panel B. Lung cancer mortality per 100,000 persons and percentage of US population aged 55 to 79 without access to a screening center within 30 miles. Mortality and accessibility scores were classified into 3 groups (low, medium, high), each based on the natural breaks method, and combined for bivariate mapping: 1) high mortality/high access, 2) high mortality/medium access, 3) high mortality/low access, 4) medium mortality/high access, 5) medium mortality/medium access, 6) medium mortality/low access, 7) low mortality/high access, 8) low mortality/ medium access, and 9) low mortality/low access. These maps highlight state-level variation in LDCT screening availability and accessibility, as well as lung cancer mortality among persons of LDCT screening age. The maps help identify areas in need of LDCT screening program creation and/or expansion, particularly in rural areas.

**Table:** 

Panel A. State Name (Including District of Columbia)	No. of Centers	Lung Cancer Mortality Per 100,000 Persons	Proportion of Population Aged 55-79 Outside 30-Mile Buffer, %
Alabama	23	55.5	31
Alaska	3	47.6	49
Arizona	22	36.1	23
Arkansas	10	60.1	52
California	109	33.4	8
Colorado	33	31.7	20
Connecticut	40	39.4	0
Delaware	20	51.1	0
District of Columbia	3	40.3	0
Florida	114	43.8	5
Georgia	53	47.7	14
Hawaii	5	32.1	17
Idaho	9	37.0	38
Illinois	55	47.6	11
Indiana	45	55.1	11
Iowa	15	46.0	34
Kansas	9	46.6	38
Kentucky	49	69.6	13
Louisiana	21	55.1	12
Maine	6	52.5	33
Maryland	49	43.2	1
Massachusetts	57	43.7	2
Michigan	54	49.8	13
Minnesota	21	39.7	21
Mississippi	9	58.8	42
Missouri	28	55.3	27
Montana	2	41.2	82
Nebraska	11	43.1	32
Nevada	16	46.6	12
New Hampshire	10	46.2	15
New Jersey	81	39.8	0
New Mexico	4	31.6	42
New York	143	40.1	5
North Carolina	64	50.7	8
North Dakota	3	40.2	78
Ohio	78	52.8	7
Oklahoma	8	56.5	34
Oregon	18	44.0	27
Pennsylvania	134	46.4	1
Rhode Island	17	48.8	0
South Carolina	30	50.4	12
South Dakota	2	43.9	74
Tennessee	34	58.6	27
Texas	101	40.6	21
Utah	3	20.0	33
Vermont	5	47.1	17
Virginia	58	45.5	14
Washington	31	42.2	22.5
West Virginia	14	60.3	29
Wisconsin	18	44.2	32
Wyoming	1	36.7	86

**Table:** 

Panel B. State Name (Including District of Columbia)	State Mortality and Access Classification	Legend Reference
Delaware	High Mortality/High Access	7
Indiana	High Mortality/High Access	7
Kentucky	High Mortality/High Access	7
Louisiana	High Mortality/High Access	7
Ohio	High Mortality/High Access	7
Alabama	High Mortality/Medium Access	8
Mississippi	High Mortality/Medium Access	8
Missouri	High Mortality/Medium Access	8
Oklahoma	High Mortality/Medium Access	8
Tennessee	High Mortality/Medium Access	8
West Virginia	High Mortality/Medium Access	8
Maine	High Mortality/Medium Access	8
Arkansas	High Mortality/Low Access	9
Florida	Medium Mortality/High Access	4
Georgia	Medium Mortality/High Access	4
Illinois	Medium Mortality/High Access	4
Maryland	Medium Mortality/High Access	4
Massachusetts	Medium Mortality/High Access	4
Michigan	Medium Mortality/High Access	4
Nevada	Medium Mortality/High Access	4
New Hampshire	Medium Mortality/High Access	4
North Carolina	Medium Mortality/High Access	4
Pennsylvania	Medium Mortality/High Access	4
Rhode Island	Medium Mortality/High Access	4
South Carolina	Medium Mortality/High Access	4
Vermont	Medium Mortality/High Access	4
Virginia	Medium Mortality/High Access	4
Alaska	Medium Mortality/Medium Access	5
Iowa	Medium Mortality/Medium Access	5
Kansas	Medium Mortality/Medium Access	5
Nebraska	Medium Mortality/Medium Access	5
Oregon	Medium Mortality/Medium Access	5
Texas	Medium Mortality/Medium Access	5
Washington	Medium Mortality/Medium Access	5
Wisconsin	Medium Mortality/Medium Access	5
Montana	Medium Mortality/Low Access	6
South Dakota	Medium Mortality/Low Access	6
California	Low Mortality/High Access	1
Connecticut	Low Mortality/High Access	1
District of Columbia	Low Mortality/High Access	1
Hawaii	Low Mortality/High Access	1
New Jersey	Low Mortality/High Access	1
New York	Low Mortality/High Access	1
Minnesota	Low Mortality/Medium Access	2
Arizona	Low Mortality/Medium Access	2
Colorado	Low Mortality/Medium Access	2
Idaho	Low Mortality/Medium Access	2
New Mexico	Low Mortality/Medium Access	2
Utah	Low Mortality/Medium Access	2
North Dakota	Low Mortality/Low Access	3
Wyoming	Low Mortality/Low Access	3

## Background

Lung cancer is the leading cause of cancer-related death among men and women in the United States, with more than 234,000 persons diagnosed yearly (1). Most diagnoses are made at a late stage when the cancer is more difficult to treat (1). Until 2011, there was not broad evidence that any one type of screening reduced lung cancer mortality. However, the National Lung Screening Trial clearly demonstrated that annual low-dose computed tomography (LDCT) can reduce lung cancer deaths by up to 20% in high-risk populations (ie, people aged 55–74 y who have a ≥30-pack-year smoking history and who, if former smokers, had quit within the previous 15 years) (2). Subsequently, in 2014, the US Preventive Services Task Force (USPSTF) (3) endorsed LDCT screening for high-risk persons, resulting in changes to the reimbursement policies of private and public insurers.

Despite endorsements and new reimbursement policies favoring LDCT screening, uptake is low among high-risk persons (3.9% based on the 2015 National Health Interview Survey) (4). In 2014, we showed that the United States had 203 active LDCT screening centers and that most were in the Northeast and the East North Central states (5). In 2015, the Centers for Medicare & Medicaid Services introduced insurance coverage and associated billing codes for LDCT screening. An updated assessment of the landscape of LDCT screening in the United States is now needed to determine the extent of geographic variation in LDCT screening availability and to identify regions for program expansion based on potential demand.

## Methods

In January 2017, we obtained the names and locations of LDCT screening centers that meet/attest to quality standards set by the Lung Cancer Alliance (ie, Screening Centers of Excellence) and/or the American College of Radiology (ie, ACR Designated Lung Cancer Screening Centers). The business addresses of each LDCT screening center was geocoded using Esri’s World Geocoding Service and ArcGIS Desktop version 10.2.

The primary geographic unit was the census block group. Block groups were classified as urban or rural areas according to 2010 Rural–Urban Commuting Area (RUCA) codes established by the US Department of Agriculture’s Economic Research Service. Each block group was assigned an urban or rural designation according to whether its centroid (ie, geographic center of a polygon) was inside the census-tract boundaries in which it was nested. RUCA codes of 1, 2, and 3 were categorized as urban, and codes 4 through 10 were categorized as rural. We used population estimates for the number of persons aged 55 to 79 to approximate the USPSTF-recommended screening age (ie, 55–80 y). We obtained population estimates at the block group level from the US Census Bureau’s Integrated Public Use Microdata Series, specifying data from the 2011–2015 American Community Survey (6).

Data on state lung cancer mortality rates (aggregated data from 2010 through 2014), extracted from the National Cancer Institute’s State Cancer Profiles website (7), were overlaid with data on LDCT screening center locations to determine whether areas of high need had equitable access to services. We measured spatial accessibility to screening by using a 30-mile Euclidean distance buffer (ie, a circle around a point 30 miles in any direction) to identify the number of persons aged 55 to 79 who reside within and outside the facility catchment area. A centroid was created for each block group and was assigned a corresponding block group population. To calculate the population residing within and outside of each buffer, we assumed that if a census block group’s centroid was inside the 30-mile buffer, then the population in that block group would have access to that screening center. We aggregated block group estimates to compute accessibility for all 50 states and the District of Columbia and by level of rurality (urban or rural). For the first map, the proportion of persons aged 55 to 79 without access to a designated LDCT screening center within 30 miles was categorized into 4 groups in equal intervals; the breaks occurred at 22%, 43%, and 65%. For a second map, lung cancer mortality and accessibility scores were classified into 3 groups by using the natural breaks method. For lung cancer mortality, these groups were classified as low (20.0 to 40.3 deaths per 100,000), medium (40.6 to 50.7 deaths per 100,000), and high (51.1 to 69.6 deaths per 100,000). For accessibility, the groups were classified as low (51%–86%), medium (20%–48%), and high (0%–17%). These categories resulted in 9 combinations of lung cancer mortality and accessibility, presented in bivariate map format. Finally, we performed a sensitivity analysis to examine accessibility based on a 30-minute driving distance.

## Main Findings

The number of designated LDCT screening centers increased from an estimated 203 in 2014 to 1,748 in early 2017. The mean number of designated LDCT screening centers per state was 34. Nine states (Alaska, Hawaii, Montana, New Mexico, North Dakota, South Dakota, Utah, Vermont, and Wyoming) and the District of Columbia had 5 or fewer designated centers.

Across all states, an average 14.9% of persons aged 55 to 79 did not have access to a designated screening center within 30 miles, and an average 28.1% did not have access within a 30-minute drive. In most states (27 states and the District of Columbia), 0% to 22% of the population aged 55 to 79 did not have access to a designated screening center within 30 miles; 66% to 86% of this population did not have access to these services within 30 miles in 4 states (North Dakota, South Dakota, Montana, and Wyoming) clustered in the upper Midwest (Panel A). In 9 states (Connecticut, Delaware, Florida, Maryland, Massachusetts, New Jersey, New York, Pennsylvania, and Rhode Island) and the District of Columbia, 95% or more of the population aged 55 to 79 had access to a designated LDCT screening center within 30 miles. Rural residents were less likely than urban residents to have access to a designated LDCT screening center within 30 miles (47.5% rural vs 93.7% urban) or a 30-minute drive (22.2% rural vs 83.2% urban).

Lung cancer mortality was highest in the eastern interior of the United States. A cluster of high mortality stretches north to south from West Virginia to Louisiana and west to east from Oklahoma to West Virginia. Despite their high rate of lung cancer mortality, Alabama, Arkansas, Mississippi, Oklahoma, Tennessee, and West Virginia had low to medium levels of access to LDCT screening. All the states along the eastern seaboard and in the Northeast, except Maine, had high access to LDCT screening (Panel B).

## Action

These maps update an assessment of the geographic variation of LDCT screening availability in the United States and extend research by using spatial proximity of persons aged 55 to 79 to designated screening centers to identify disparities in access (5,8). Findings indicate that although the number of designated LDCT screening centers increased by more than 8 times since 2014, pronounced disparities in the distribution of centers exist, particularly between rural and urban areas. This disparity in access is concerning, given the large proportion of high-risk persons in rural areas. We hope that the geographic patterns illustrated in these maps stimulate further research into ways to improve equitable access to high-quality screening services in high-need regions.
